# Influence of drinking water containing *Aloe vera* (*Aloe barbadensis* Miller) gel on growth performance, intestinal microflora, and humoral immune responses of broilers

**DOI:** 10.14202/vetworld.2016.1197-1203

**Published:** 2016-11-05

**Authors:** Meisam Shokraneh, Gholamreza Ghalamkari, Majid Toghyani, Nasir Landy

**Affiliations:** 1 Young Researchers and Elite Club, Isfahan (Khorasgan) Branch, Islamic Azad University, Isfahan 8155139999, Iran; 2 Department of Animal Science, Isfahan (Khorasgan) Branch, Islamic Azad University, Isfahan 8155139999, Iran

**Keywords:** *Aloe vera* gel, broiler, ileal microflora, immune responses, performance

## Abstract

**Aim:**

The risk of bacteria resistance to specific antibiotics possibly by continuous subtherapeutical administration of antibiotic growth promoters (AGPs) in poultry feed led to a ban on the use of AGP in poultry production. As a result of this ban, alternative substances for poultry growth promotion and disease prevention are being investigated, among which phytogenic and herbal products have received increased attention as natural additives because they have been accepted by consumers as natural additives. The effect of water supplementation of *Aloe vera* (AV) as an AGP substitute on performance, intestinal microflora, and immune responses of broilers.

**Materials and Methods:**

The five experimental treatments were allocated to four replicates. The following treatments were applied (1) a basal broiler diet (C) and normal drinking water, (2) 0.5% AV gel in drinking water, (3) 0.75% AV gel in drinking water, (4) 1% AV gel in drinking water, and (5) diet C supplemented with flavophospholipol at 4.5 mg/kg and drinking normal water. Vaccines against influenza disease and sheep red blood cell (SRBC) were administrated to immunological stimuli. The populations of *Lactobacilli* spp. and coliforms were enumerated in ileum.

**Results:**

Body weight of broilers supplemented with different levels of AV increased compared with control group (p<0.05). Birds supplemented with antibiotic had the best feed-to-gain ratio (F:G) in different periods. Supplementation of 0.5% and 0.75% AV improved F: G entire experimental period compared with control group (p<0.05). Coliform bacteria were reduced in broilers supplemented with different levels of AV or antibiotic (p<0.05). The *Lactobacilli* spp. population in birds supplemented with 0.75%, 1% AV or antibiotic significantly was higher than other groups (p<0.05). Supplementation with 1% AV led to greater antibody titers against SRBC compared with other groups (p<0.05).

**Conclusion:**

These findings demonstrated a possibility of supplementing broiler drinking water with 1% AV gel as an alternative for AGP substitution.

## Introduction

So far, subtherapeutic dosage of antibiotics has been used more than 50 years in poultry nutrition to promote growth performance and prevent diseases [1-4]. However, continuous use of in-feed antibiotics is suspected to result in common problems such as increasing resistance of pathogens to antibiotics [5], accumulation of antibiotic residues in animal products and the environment [6,7], and imbalance of normal microflora [8]. Thus, efforts have been made to substitute antibiotic growth promoters (AGPs) with possible alternative growth promoters. Phytogenic and herbal products have received increased attention as natural additives in recent years because they have been accepted by consumers as natural additives [9-12].

*Aloe vera* (AV) (*Aloe barbadensis* Miller) is a well-known medicinal herb and it has been used for commercial and therapeutic properties in many parts of the world [13]. It is a tropical plant of the genus *Aloe* and belongs to the Liliaceae family. AV gel contains compounds with proven antibacterial, antiviral, antifungal, antioxidant, anti-inflammatory, anti-diabetic, immunomodulatory, and wound healing properties [14]. AV gel contains acemannan, which has been identified as the primary polysaccharide [14,15]. Polysaccharides can affect the humoral immune response and cellular immunity [16]. Studies showed that acemannan is able to activate of macrophages to release inflammatory cytokines such as interleukin-1 (IL-1), IL-6, and tumor necrosis factor-α (TNF-α) [17,18]. AV gel has demonstrated antimicrobial properties against a wide range of pathogenic bacteria such as *Staphylococcus aureus* and *Escherichia coli* [19,20].

Darabighane and Nahashon [21] observed the beneficial influence of AV gel on intestinal microflora and immunity in broiler chickens. Furthermore, Feng *et al*. [22] reported positive effects of AV relative weight of lymphoid organs of broilers. Despite these findings, few studies have evaluated efficiency of AV on performance, intestinal microflora, and immune responses in broilers. The aim of this trial was to further enhance our knowledge and examine the effects of AV gel on broiler growth performance and other important biomarkers for broiler development and health such as carcass characteristics, ileal microflora, and humoral immunity when it is supplemented in drinking water.

## Materials and Methods

### Ethical approval

The animals were rared in accordance with the U.S. National Institutes of Health Guide for the Care and Use of Laboratory Animals. Furthermore, all procedures complied with the ethical guidelines of the Isfahan University’s Ethical Committee, Islamic Azad University, Isfahan Branch, Iran (approval ref no. 2014-854).

### Plant material

Fresh *Aloe* leaves were collected from botanical garden for the extraction of gel. The leaves were cleaned with water and the *Aloe* gel was extracted from the leaf manually by making a cut. Latex of the leaf was removed, and gel was collected in a beaker. A 10% (w/v) concentrated infusion was prepared by taking 100 g of fresh gel in a glass bottle, and a liter of hot boiled distill water was poured on it. The bottle was shacked for 5-7 min to ensure thorough mixing and was then kept for 6-8 h at room temperature before use. The chemical characterization of the AV gel is provided in Table-1.

**Table 1:** Chemical characterization of AV gel.

Parameters	Content, %
Moisture	98.5
pH^1^	4.53
Glucomannan	0.05
Polimanosa	0.72

1 pH is dimensionless. AV=*Aloe vera*

### Birds and experimental treatments

Procedures performed in this trial were reviewed and approved by the Animal Care Committee of University of Isfahan. 240 1-day-old as hatched broilers (Ross 308) were purchased from a local hatchery. Broilers were individually weighed and randomly allotted to 5 experimental treatments for 6 weeks. Each treatment had 48 broilers which were arranged in 4 replicates of 12 broilers each. The following treatments were applied: (1) Basal broiler diet (C) and normal drinking water, (2) 0.5% AV gel in drinking water, (3) 0.75% AV gel in drinking water, (4) 1% AV gel in drinking water, and (5) basal diet supplemented with flavophospholipol at 4.5 mg/kg and normal drinking water. The broilers received a corn-soybean meal basal diet (Table-2) formulated to meet or exceed the nutrient requirements of Ross 308 [23]. The chicks were fed a starter diet from 0 to 14 days, a grower diet from 15 to 28 days, and finisher diet from 29 to 42 days. All diets were in mash form. There was no coccidiostat added to the basal diet. Each replicate was assigned to a clean floor pen (120 cm×120 cm), and birds were raised on wood shavings. Ventilation was provided by negative pressure with fans. Heat was provided by gas-fired brooders; water and feed were offered *ad libitum*. The lighting program consisted of a period of 23 h light and 1 h of darkness. The ambient temperature in the experimental house was maintained at 32°C during the 1^st^ week and gradually decreased by 3°C/week, and finally fixed at 22°C thereafter.

**Table 2:** The ingredient and calculated composition of basal starter, grower, and finisher diets (as-fed basis).

Item	Starter (days 1-14)	Grower (days 15-28)	Finisher (days 29-42)
Ingredient, g/kg			
Corn	537.3	533	561.5
Soybean meal (44% CP)	400	396	370
Soybean oil	20	35	35
Di-calcium (22% Ca), phosphate (17% P)	19.3	17.1	15.6
CaCO_3_	10.5	8.7	8.5
NaCl	3.5	3	3
Trace mineral premix^1^	2.5	2.5	2.5
Vitamin premix^2^	2.5	2.5	2.5
DL-methionine	3.1	2	1.4
L-lysine	1.3	-	-
Calculated composition, g/kg			
Metabolizable energy, kcal/kg	2870	2980	3000
Crude protein	221	220	210
Calcium	8.6	7.5	7
Available phosphorus	4.9	4.4	4.1
Methionine+cysteine	10.1	8.9	8
Lysine	13.3	11.9	11.3
Threonine	8.3	8.3	6.3
Tryptophan	3.2	3.2	3

1 Provided the following per kg of diet: Mg - 56 mg; Fe - 20 mg; Cu - 10 mg; Zn - 50 mg; Co - 125 mg; I - 0.8 mg.

2 Provided the following per kg of diet: Vitamin A - 10,000 IU; vitamin D_3_-2000 IU; vitamin E - 5 IU; vitamin K - 2 mg; riboflavin - 4.20 mg; vitamin B_12_-0.01 mg; pantothenic acid - 5 mg; nicotinic acid - 20 mg; folic acid - 0.5 mg; choline - 3 mg

### Performance and carcass components

Body weights (BW) of broilers were determined at 1, 14, 28, and 42 days of age. Daily weight gain, daily feed intake (DFI), and daily water consumption were recorded in different growth periods, and the feed-to-gain (F:G) was calculated. All mortalities were removed from the pens and recorded during the experiment. At 42 days of age, two birds per replicate were chosen, based on the average weight of the group and slaughtered through cutting carotid arteries and partial slicing of the neck. Carcass yield was calculated by dividing eviscerated weight by live weight. The proventriculus, gizzard, heart, liver, and abdominal fat were removed, weighed, and calculated as percentages of live weight.

### Microbial analysis

Ileal contents (from Meckel’s diverticulum to the ileocecal junction) were collected from the slaughtered birds. The samples were placed in ice and thereafter sent to the microbiology laboratory for bacteriological analysis. 1 g sample was taken from each bird’s ileal contents and blended with 9 mL of sterilized physiological saline solution and homogenized by a shaker. These suspensions were serially diluted to 10^−7^ using sterilized physiological saline solution. Appropriate dilutions were subsequently plated on duplicate selective agar media for enumeration of target bacterial groups. The selective media used for microbial culture were Macconkey agar (Merck) for coliforms and de Man Rogosa Sharp agar (CM0361, Oxoid Limited Kingdom) for *Lactobacilli* spp. The selective media for the microbial counts of coliform were incubated aerobically at 37°C for 24 h. The agar plates for the counts of *Lactobacillus* were incubated in aseptic anaerobic jars at 37°C for 48 h. The total colony count was then calculated as the number of colonies by reciprocal of the dilution. The microbial counts were expressed as log_10_ colony forming units per gram (log_10_ cfu/g) of sample [24].

### Immunity

The commercially available oil-adjuvant injectable emulsion against Newcastle Disease virus (NDV), and avian influenza virus (AIV) was used (H9N2 subtype) for vaccinating broiler chicks and it was injected subcutaneously at 9 days of age. At 28 days of age, 2 birds per replicate were randomly chosen, and blood samples were collected from brachial vein and centrifuged at 3000 g for 10 min to obtain serum (SIGMA 4-15 Lab Centrifuge, Germany). Serum antibody titers against AIV were measured by the hemagglutination inhibition test.

At 25 days of age, 8 birds from each treatment group were inoculated via the brachial vein with 1 mL of a 1% suspension of sheep red blood cell (SRBC) prepared in phosphate-buffered saline. Blood samples were collected from challenged birds 6 days after and anti-SRBC titers were measured by the microtiter method of Wegmann and Smithies [25]. All titers were expressed as the log_2_ of the reciprocal of the highest dilution given visible hemagglutination.

### Statistical analysis

The data were subjected to analysis of variance procedures appropriate for a completely randomized design using the general linear model procedures of SAS (SAS Inst., Inc., Cary, NC, USA). Means were compared using Tukey’s HSD test. Statistical significance was determined at p<0.05.

## Results

### Performance and carcass traits

Data on performance indices are presented in Table-3. Treatments failed to induce any marked effect on BW of broilers at 14 days of age. AV supplementation at different levels and the antibiotic supplementation significantly (p<0.05) increased BW of broilers at 28 and 42 days of age. Broilers receiving different levels of AV gel had higher daily water consumption compared with other groups during growth period (p<0.05), but in the other periods, it was not affected. Supplementing drinking water with 1% AV gel significantly (p<0.05) increased DFI during the experimental period relative to the control. Water supplemented with different levels of AV significantly (p<0.05) enhanced F:G and DFI during grower period. Broilers receiving flavophospholipol had lower F:G compared with broilers receiving basal diets and normal drinking water during starter, grower, finisher periods, and entire experimental period (p<0.05). Water supplemented with 0.75% and 1% AV significantly (p<0.05) improved F:G during the entire experimental period compared with broilers fed basal diet and normal drinking.

**Table 3:** Effect of antibiotic supplementation in the diet and AV supplementation via drinking water on performance indices of broiler chicks.

Item	Experimental treatments^1^					SEM

	Control	0.5% AV	0.75% AV	1% AV	Antibiotic	
BW, g						
Day 14	368	378	387	381	384	6.5
Day 28	945^b^	1043^a^	1072^a^	1086^a^	1075^a^	21.9
Day 42	2190^b^	2355^a^	2338^a^	2417^a^	2433^a^	46.1
Daily feed intake, g/day						
Days 1-14	36.7^ab^	35.5^b^	36.8^ab^	38.3^a^	36.7^ab^	0.69
Days 15-28	88.9^b^	95.8^a^	95.8^a^	96.1^a^	92.6^ab^	1.97
Days 29-42	168	179.3	172.4	179.9	174.5	3.99
Days 0-42	98.1^b^	103.5^ab^	101.8^ab^	104.9^a^	101.5^ab^	1.78
Daily water consumption, g/day						
Days 1-14	75.5	77.8	76.7	77.4	79.9	1.57
Days 15-28	187^b^	207.4^a^	209.4^a^	208.6^a^	198.8^ab^	5.12
Days 29-42	352.9	377	358	385	360.9	12.67
Days 1-42	205.5	221.2	215.1	223.9	213.5	5.6
F:G, g:g						
Days 1-14	1.71^a^	1.59^c^	1.61^bc^	1.70^ab^	1.61^bc^	0.027
Days 15-28	2.18^a^	1.99^b^	1.96^bc^	1.90^bc^	1.87^c^	0.031
Days 29-42	2.01^a^	2.04^a^	2.04^a^	2.02^a^	1.92^b^	0.022
Days 1-42	2.01^a^	1.97^ab^	1.95^b^	1.94^b^	1.86^c^	0.014

a-c Means in the same row not sharing a common superscript differ (p<0.05).

1 Control: Basal broiler diet and normal drinking water; 0.5% AV: 0.5% AV gel in drinking water; 0.75% AV: 0.75% AV gel in drinking water; 1% AV: 1% AV gel in drinking water; Antibiotic: Basal diet+4.5 mg flavophospholipol/kg. AV=*Aloe vera*; SEM=Standard error of mean

Table-4 shows carcass and relative organ weights as a percentage of live weight at slaughter. Carcass yield, abdominal fat, liver, gizzard, heart, and proventriculus weights were not significantly affected by the dietary and water treatments, though carcass yield tended to increase in broilers supplemented with 1% AV via drinking water or antibiotic in the diet (p>0.05).

**Table 4:** Effect of experimental treatments on carcass yield and internal relative organ weight of broilers at 42 days of age.

Relative organ weight, %	Experimental treatments^1^					SEM

	Control	0.5% AV	0.75% AV	1% AV	Antibiotic	
Carcass	71.5	71.2	71	72.1	72.5	0.516
Abdominal fat	1.10	1.27	1.23	0.99	1.22	0.086
Liver	2.13	2.11	2.23	2.35	2.34	0.078
Gizzard	2.39	2.50	2.38	2.36	2.47	0.087
Heart	0.54	0.55	0.53	0.54	0.58	0.026
Proventriculus	0.37	0.40	0.39	0.36	0.37	0.017

AV=*Aloe vera*, SEM=Standard error of mean

### Intestinal microflora

Supplementing drinking water with different levels of AV or antibiotic in the diet significantly reduced concentration of coliforms, relative to control (Table-5). Maximum concentration of *Lactobacilli* spp. population reached in broilers supplemented with drinking water containing 1% AV gel. An improved total *Lactobacillus* population was noted in groups supplemented with 0.75% and 1% AV gel as compared with control and 0.5% AV gel (p<0.05). Total *Lactobacillus* population significantly increased in broilers fed diets containing 4.5 mg flavophospholipol per kg diet (p<0.05).

**Table 5:** Effect of experimental treatments on ileum bacteria populations of broiler chicks at 45 days of age.^1^

Items	Experimental treatments					SEM

	Control	0.5% AV	0.75% AV	1% AV	Antibiotic	
*Lactobacilli* spp.	5.84^c^	5.89^c^	6.33^b^	7.69^a^	6.32^b^	0.098
Coliforms	5.11^a^	4.10^bc^	3.97^c^	3.89^c^	4.07^bc^	0.321

a-c Means in the same row not sharing a common superscript differ (p<0.05).

1 Results are given as means of groups (n=4). AV=*Aloe vera*, SEM=Standard error of mean

### Immune responses

The effect of experimental treatments on antibody titers against influenza virus at day 28 and SRBC at day 30 are presented in Table-6. The treatments had no effect on antibody titer against AIV. Broilers supplemented with 1% AV via drinking water showed an improved antibody titer against SRBC compared with those supplemented with basal diet and normal drinking water or basal diet containing antibiotic (p<0.05).

**Table 6:** Effect of antibiotic supplementation in the diet and AV supplementation via drinking water on antibody titers against Newcastle and influenza viruses at 28 days of age and SRBC at 31 days of age.

Item	Experimental treatments					SEM

	Control	0.5% AV	0.75% AV	1% AV	Antibiotic	
Influenza (log_2_)	3.37	1.75	2.75	2.62	2.00	0.76
SRBC (log_2_)	7.25^b^	7.75^ab^	7.75^ab^	8.37^a^	6.75^b^	0.34

a, b Means in the same row not sharing a common superscript differ (p<0.05). AV=*Aloe vera*, SEM=Standard error of mean, SRBC=Sheep red blood cell

## Discussion

In this study, broilers receiving antibiotic had higher BW, F:G and total *Lactobacillus* population and lower concentration of coliforms, relative to broilers receiving basal diets and normal drinking water. Antibiotics may control and inhibit colonization of pathogenic and nonpathogenic species of bacteria in gastrointestinal tract of poultry [26]. A modulated intestinal microflora of broilers could lead to better nutrient utilization, resulting in an increased BW and improved F:G [27]. Results of this experiment indicated that supplementing drinking water with AV gel could induce favorable influences on growth performance and intestinal microflora of broilers. As AV gel has been reported to contain compounds with proven antibacterial [19], antioxidant [28], and immunostimulatory [17] properties; an increase in growth performance was anticipated. Furthermore, AV gel contains several beneficial ingredients including vitamins, minerals, enzymes, organic acids, and carbohydrates which could improve performance criteria of broilers [29]. The results of this trial indicated that supplementation of AV gel to drinking water seem to have an affirmative influence on BW via higher DFI. Similar results were found in the research conducted by Olupona *et al*. [30]. They investigated the effect of supplementation of AV gel in drinking water on broiler performance and observed that the final BW improved via higher DFI. In another study in broilers, Darabighane *et al*. [31] reported that broilers fed dietary supplemented with 1.5%, 2% and 2.5% AV gel indicated higher DFI and BW than those fed basal diets. They also observed that 2% AV gel significantly enhanced villus height to crypt depth ratio in comparison with antibiotic group. Odo *et al*. [32] and Mmereole [33] also reported that inclusion of AV leaves in broiler feed increased BW compared with the control. Likewise, Changkang *et al*. [34] indicated that 600 mg of AV gel water extract added in broiler feed resulted in significant increase of BW in the 3^rd^ and 6^th^ weeks, in comparison with the control. In contrast, Mehala and Moorthy [35] assessed 0.1% and 0.2% AV powder and reported no effects on DFI, BW, and F:G in broilers.

In the present trial, the affirmative impact of AV gel on the F:G can be related to more balanced biota population in the chicks’ gut. As medicinal plants and their properties may control and inhibit colonization of pathogenic and nonpathogenic species of bacteria in gastrointestinal tract of poultry. This may lead to a more efficient use of nutrients, resulting in increased growth and improved F:G [27]. The effect of AV gel on microflora composition is related to the polysaccharides specially acemannan which behave similar to prebiotics [21]. Furthermore, acemannan has been reported to have indirect antimicrobial activity by its ability to stimulate macrophages [36]. AV gel has been shown to have bactericidal activity against a wide range of Gram-negative and positive bacteria such as *S. aureus*, *Streptococcus pyogenes*, *E. coli*, and *Salmonella Typhi* [37]. Lin *et al*. [38] indicated that AV gel powder, *Aloe* polysaccharides, and acemannan could significantly reduce the cecal contents of *E. coli* and increased cecal bifidobacteria and lactic acid bacteria concentration. The reduced count of coliforms observed in the broilers received AV gel was related to the increased counts of *Lactobacilli*. In fact, *Lactobacilli* compete with pathogens for nutrients and sites of intestinal adhesion [39]. Another beneficial activity of *Lactobacilli* is synthesis of bacteriocin [40]. Lactic acid and acetic acid, which are formed by the fermentation of *Lactobacilli*, could limit the growth and colonization of pathogenic bacteria in chicks’ gut [39]. Results of the present research confirmed those obtained by Heggers *et al*. [19], Ferro *et al*. [41], Shilpakala *et al*. [20], and Saritha *et al*. [42].

In this trial, carcass yield and internal organ weight were not markedly affected by experimental treatments. Our findings are consistent with those reported by Silalahi *et al*. [43] who did not find any differences among the control treatment and those containing dried or fresh AV gels on carcass traits. However, in another trial, the percentage of carcass and breast were affected by AV juice application through drinking water [30]. In accord to our results, several studies have shown that addition of antibiotic to the broiler’s diets and had not any marked effect on carcass traits [1,44,45].

So far, antibody responses have been used as measures of humoral immune status of birds. The treatments had no effect on antibody titer against AIV. The reason might be related to the type of applied vaccine which was inactivated and/or vaccination date. Antibody titer against SRBC increased in the group treated with 1% AV in drinking water compared with those on the control and flavophospholipol diets. Similarly, Akhtar *et al*. [46] reported significantly higher anti-SRBC antibody (total immunoglobulins [Igs], IgG and IgM) titers in chickens supplemented with ethanolic extract of AV as compared with the control group. Results of another trial revealed significantly higher humoral immune responses against SRBC and NDV in chickens supplemented with 2.5% AV gel in diet [47]. Likewise, Valle-Paraso *et al*. [48] demonstrated significant increase in NDV antibody titers and absolute counts of monocytes, lymphocytes, and heterophils in broilers received 2% extract of AV gel in drinking water compared with control. Several studies in broilers have also confirmed that *Aloe* and its extract can enhance immune response and specific antibody levels [34,38,49]. Acemannan, a major polysaccharide present in AV gel, appeared to be able to activate macrophages via binding to mannose receptors. Acemannan activates macrophages to release cytokines such as IL-1, IL-6 and TNF-α, thus leading to an increase in the number and function of cytotoxic T-cells [17,50]. The acemannan, was also shown to increase nitric oxide production by macrophages, and upregulate phagocytic and candidacidal activities [18,51]. The immune enhancing effect of acemannan is also mentioned to be due to the induced maturation of immature dendritic cells, which are the important accessory cells for the initiation of primary immune response [52]. Moreover, Chinnah *et al*. [53] found that acemannan has strong adjuvant properties in vaccination against avian viral diseases such as NDV and Marek’s disease.

## Conclusion

These findings demonstrated a possibility of supplementing broiler drinking water with 1% AV gel as an alternative for AGP substitution.

## Authors’ Contributions

MS, MT, and GG have designed the plan of work. MS and NL carried out the laboratory work and analyzed the results. MS and NL drafted and revised the manuscript. All authors read and approved the final manuscript.
